# 

**Published:** 1909-05-08

**Authors:** 


### BOOKS RECEIVED.

Cassell and Co., Ltd.

"A Manual of Operative Surgery." Third edition. By Sir Frederic Treves, G.C.V.O., C.B., LL.D., F.R.C.S., and Jonathan Hutchinson, F.R.C.S.

"A Handbook for Midwives and Maternity Nurses." By Comyns Berkeley, B.A., M.B., B.C., etc.

Henry Frowde, Hodder, and Stoughton.

"Suture of Arteries." By E. Archibald Smith, M.B., Ch.B., F.R.C.S.

C. Mitchell and Co.

"Newspaper Press Directory, 1909."

"Heart Disease Graphic Methods." By J. Hay, M.D., M.R.C.P.

Baillière, Tindall, and Cox.

"Operative Nursing and Technique." By Charles P. Childe, B.A., F.R.C.S.

"Aids to Medicine." By Bernard Hudson, M.D., M.R.C.P.

THE
### GRADUATES' MEDICAL SCHOOLS.

DIARY FOR THE WEEK MAY 10 TO MAY 14.

### MEDICAL GRADUATES' COLLEGE AND POLYCLINIC, 22 Chenies Street, W.C.

**At 5.15 p.m.**

**May 10,** Dr. T. D. Savill, **Treatment of Neurasthenia.**

**May 11,** Mr. Thomson Walker, **Diagnosis and Treatment of Malignant Disease of the Bladder.**

**May 12.** Dr. C. O. Hawthorne, **The Scope and Purpose of the British Pharmacopœia.**

**May 13,** Dr. F. J. Wethered, **The Treatment of Chronic Heart Disease, especially by Mechanical Methods.**

### THE THROAT HOSPITAL, Golden Square, W.

**At 5.30 p.m.**

**May 10,** Mr. Parker, **Examination of the Ear and Hearing Powers.**

**May 13,** Mr. Parker, **Affections of the External Auditory Canal.**

### NORTH-EAST LONDON POST-GRADUATE COLLEGE, Prince of Wales's General Hospital, Tottenham, N.

**At 4.30 p.m.**

**May 13,** Mr. W. Edmunds, **Knots, Ligatures, and Sutures.**

### THE POST-GRADUATE COLLEGE, West London Hospital, Hammersmith, W.

**At 10 a.m.**

**May 10 and 13,** Surgical Registrar, **Demonstration.**

**May 14,** Medical Registrar, **Demonstration.**

**At 12 noon.**

**May 10,** Dr. Bernstein, **Pathological Demonstration.**

**At 12.15 p.m.**

**May 12 and 15,** Dr. Pritchard, **Practical Medicine.**

**At 5 p.m.**

**May 10 and 13,** Dr. Davis, **Diseases of Throat, Nose, and Ear.**

**May 11,** Dr. Moullin, **Gynæcology.**

**May 12,** Dr. Beddard, **Medicine—I.**

**May 14,** Dr. Low, **Plague.**

### THE DEATH RATE FOR THE FIRST QUARTER.

A few observations suggested by the Death Rate Diagram for the first quarter of the current year, which we published last week, may not be out of place in the present issue.

The year 1909 opened well from a health point of view, and for several weeks was full of promise. Following upon one of the healthiest years on record, the month of January sustained to the full the highly satisfactory vital statistics of 1908.

The death-rate of the great towns in February did not exceed the septennial average, but with March a marked excess over the mean rate was to be observed. The mortality during the last month of the quarter was so high, in fact, as more than to compensate for the saving effected during January and February, and the second quarter of the year thus starts with the mean annual death-rate materially below that of the current year.

The March mortality was considerably augmented by the prevalence of influenza. It is not the diseases of high fatality which swell the death-rate so much as those of widespread prevalence. Measles, whooping-cough, zymotic diarrhœa, influenza, have none of them a high degree of fatality. They are, comparatively speaking, and in the great majority of persons attacked, trivial affections, in which the question of danger to life barely arises; yet because of the large number of susceptible persons, and among them the high degree of probability of attack, large numbers of the population are seized in times of epidemic prevalence. And an extensive morbidity of this sort counts for more in the mortality bills than does the lesser prevalence of more serious affections.

When influenza is epidemic comparatively few people escape wholly the incidence of the disease. It may be a passing lumbago, a transient headache, a twinge of muscular rheumatism, an indefinable sense of malaise that alone betrays the presence of the infection, but the universal diffusion of the poison exposes the weak places in which may be styled the communal resistance, and this is expressed in a mortality-rate such as is exhibited above in the curve for March of the 76 great towns.

Superimposed upon the normal mortality, deaths from the commonly recurring epidemics of minor diseases disturb the seasonal curve and lend to particular years those undesirable features represented in steep ascents and towering pinnacles which are the sport of agencies not yet within the grip of preventive control.

In the University of Oxford on May 4, the congregation approved a statute which had been proposed for the establishment of a Diploma in Ophthalmology.

The next session of the General Medical Council will commence on Tuesday, May 25. The President, Principal Sir Donald MacAlister, K.C.B., will take the chair at 2 p.m.

The President and Council of the Medical Society of London will give a conversazione at the Society's rooms at 11 Chandos Street, W., on Monday, May 17. There will be a reception by the President at 8.30 p.m., and at 9 p.m. an oration by Dr. H. D. Rolleston on the Classification and Nomenclature of Diseases, with remarks on Diseases due to Treatment.

